# Supplementary material for: A retrospective analysis of prenatal genetic results in fetal hydronephrosis
Source: PLoS One. 2025 May 22;20(5):e0324734. doi: 10.1371/journal.pone.0324734 (PMC12097554; doi:10.1371/journal.pone.0324734)
Supplement: Attachment — Submitted filename: Response Letter20250323.docx [file pone.0324734.s002.docx]

Response Letter

Dear Editor,

We quite appreciate your favorite consideration and the reviewers’insightful comments concerning our manuscript entitled “A Retrospective Analysis of Prenatal Genetic Results in Fetal Hydronephrosis” (ID：PONE-D-24-33836).

We have carefully studied these requirements and made the revisions. We hope that you will approve of these modifications. Thank you for your comments. The revised parts are as follows:

**Journal Requirements:**

1. We note your Data Availability statement as follows:

The data that support the findings of this study are not openly available dueto reasons of sensitivity and are available from the corresponding author upon reasonable request. Data are located in controlled access data storage at National Genomics Data Center(https://ngdc.cncb.ac.cn/).

OMIX ID: OMIX005796.Shared URL:https://ngdc.cncb.ac.cn/omix/view/OMIX005796

In this instance it seems there may be acceptable restrictions in place that prevent the public sharing of your minimal data. PLOS requires that the minimal data for your study be made available upon request in this case. However, in line with our goal of ensuring long-term data availability to all interested researchers, PLOS’ Data Policy states that authors cannot be the sole named individuals responsible for ensuring data access (http://journals.plos.org/plosone/s/data-availability#loc-acceptable-data-sharing-methods).

Data requests to a non-author institutional point of contact, such as a data access or ethics committee, helps guarantee long term stability and availability of data. Providing interested researchers with a durable point of contact ensures data will be accessible even if an author changes email addresses, institutions, or becomes unavailable to answer requests.

Before we proceed with your manuscript, please address the following prompts:

If there are ethical or legal restrictions on sharing a de-identified data set, please explain the restrictions in detail (e.g., data contain potentially identifying or sensitive patient information) and who has imposed them (e.g., a Research Ethics Committee or Institutional Review Board, etc.). Please also provide non-author contact information (phone/email/hyperlink) for a data access committee, ethics committee, or other institutional body to which data requests may be sent. If applicable, please also provide any necessary information which interested researchers would need when requesting access to data in order to obtain the minimal data set for your study.

We will update your Data Availability statement on your behalf to reflect the information you provide.

We've returned your manuscript to your account. Please resolve these issues and resubmit your manuscript within 21 days. If you need more time, please email the journal office at plosone@plos.org. We are happy to grant extensions of up to one month past this due date. If we do not hear from you within 21 days, we will withdraw your manuscript.

Please log on to PLOS Editorial Manager at https://www.editorialmanager.com/pone/ to access your manuscript. You will find your manuscript in the 'Submissions Sent Back to Author' link under the New Submissions menu. Be sure to remove your previous manuscript file if you are uploading a new file in response to these requests. After you've made the changes requested above, please be sure to view and approve the revised PDF after rebuilding the PDF to complete the resubmission process.

We are requesting these changes to comply with the PLOS ONE submission guidelines (https://journals.plos.org/plosone/s/submission-guidelines). Please note that we won't send your manuscript for review until you have resolved the above requests.

We have made the necessary adjustments strictly in accordance with the requirements of PLOS ONE. As the data involve human genetics, they need to be publicly available only after being filed in the National Human Genetic Resources System. Currently, we have successfully completed the filing. The data can be downloaded through the above - mentioned website. Meanwhile, during the data verification, we found that one case was under - uploaded, and we have now completed the supplementation.

The supporting data of this study are stored in the controlled - access data repository at National Genomics Data Center(https://ngdc.cncb.ac.cn/).

OMIX ID: OMIX005796.Shared URL:https://ngdc.cncb.ac.cn/omix/view/OMIX005796;

OMIX ID: OMIX009450.Shared URL:https://ngdc.cncb.ac.cn/omix/view/OMIX009450.

Thank you and the review again for your help, and hope that the correction will meet with approval.

Kind regards,

Keqin jin
